# Tunable replica symmetry breaking in random laser

**DOI:** 10.1515/nanoph-2022-0757

**Published:** 2023-01-23

**Authors:** Jiangying Xia, Xiaojuan Zhang, Kaiming Zhou, Lin Zhang, Erlei Wang, Wenyu Du, Jiajun Ma, Siqi Li, Kang Xie, Benli Yu, Junxi Zhang, Zhijia Hu

**Affiliations:** Laboratory of Optical Fibers and Micro-nano Photonics, Anhui Province Key Laboratory of Measuring Theory and Precision Instrument, School of Instrument Science and Opto-Electronics Engineering, Hefei University of Technology, Hefei, 230009, Anhui, P. R. China; Information Materials and Intelligent Sensing Laboratory of Anhui Province, Key Laboratory of Opto-Electronic Information Acquisition and Manipulation of Ministry of Education, School of Physics and Opto-Electronic Engineering, Anhui University, Hefei, 230601, Anhui, P. R. China; Aston Institute of Photonic Technologies, Aston University, Birmingham B4 7ET, UK; School of Mechanical and Electrical Engineering, ZhouKou Normal University, Zhoukou, Henan 466000, P. R. China; State Key Laboratory of Environmental Friendly Energy Materials, School of Material Science and Technology, Southwest University of Science and Technology, Mianyang, Sichuan 621000, P. R. China; School of Opto-Electronic Engineering, Zaozhuang University, Zaozhuang, 277160, Shandong, P. R. China

**Keywords:** random laser, structure and temperature, tunable replica symmetry breaking

## Abstract

Replica symmetry breaking (RSB) has been widely recognized as a statistical analysis approach to understand the disorder and nonlinear interactions in complex systems ranging from atoms to the cosmic scale. However, it is challenging to analyze the nonlinear optical characteristics of random laser (RL) in disordered gain medium via RSB due to the lack of a general RSB-based statistical analysis framework. In this work, we report the tunable RSB in polymer fiber RL, where the effects of temperature and different structures on RSB are investigated experimentally and theoretically. It experimentally proves that RSB in RL is not robust, and disorder and temperature are responsible for tunable RSB in RL, which contributes to the improvement of the statistical analysis framework for investigating the optical principles of RL using RSB. And the finding of the tunable RSB allows to investigate the dynamical differences for various RL systems, which broadens the directions for the use of spin-glass theory to explore the physical mechanism of RL.

## 1 Introduction

Spin-glass theory has been widely recognized as one of the fundamental physical method to describe the complex physical meaning of different fields of research [1–3], such as, condensed matter, ecological community and random photonics. Spin-glasses are similar to general magnetic systems at high temperatures, exhibiting a paramagnetic phase. As the temperature drops below a certain value, spin-glass phase is exhibited and the ‘spin’ orientations in the system become disorder. G. Parisi [4] described the spin-glasses by an order parameter in the framework of the replica theory in 1979, i.e., replica symmetry breaking (RSB) and defined the order parameter for spin-glasses by a clear physical method in 1983 [5]. So far, RSB has become a paradigm for quantitatively analyzing the interaction of disorder and fluctuation in complex systems [6, 7]. In 2006, L. Angelani et al. [3] introduced spin-glass theory to RL. In 2015, the first experimental evidence of the RSB in solid-state RL system was reported by N. Ghofraniha et al. [7], in which the spectral intensity fluctuation overlap (IFO) is defined to as an analog to the Parisi overlap parameter (i.e., order parameter). According to the spin-glass theory, RSB defined as the transition from the paramagnetic phase to the spin-glass phase illustrates that identical systems under identical conditions may reach different states. In the fields of random photonics, especially in RL, the interactions between disorder and nonlinearity can be characterized by the statistical mechanism of RSB [8–10]. It reveals the nonlinear interactions between RL quasimodes frustrated by disorder [7]. In 2016 [11], S. Basak et al. showed that the RL system can enters the RSB regime at a narrow range around the threshold, where the phase state in the system will transfer to spin-glass phase from paramagnetic phase. In the same year [12], a phase transition from the paramagnetic phase to the spin-glass phase was observed in the modified colloidal RL by P. I. Pincheira et al., who recognized the RSB as a sign of colloidal-based RL threshold. However, we prove experimentally that RSB is not robust in the polymer fiber random laser (PFRL) in this work. It indicates that RSB is not suitable to be recognized as a marker of PFRL threshold. As a result, identifying the key factors influencing RSB in RL has become the biggest challenge to investigate the details of this statistic behavior and the rich physics involved [13].

The phase transition from paramagnetic phase to spin-glass phase has been observed around the RL threshold with increasing pump energy, according to a widely research on the RSB mechanism that has been conducted in a variety of disordered systems [14–16]. It is worth noting that the RL system belongs to the inverse temperature system, where high pump energy corresponds to low temperature and low pump energy to high temperature [1, 7]. It demonstrates that the pump energy is used to replace the temperature variable in RL. However, under a fixed pump energy, the effect of ambient temperature on RSB in RL has not been experimentally investigated in detail. According to the spin-glass theory in RL, localized modes with various characteristic lengths (determined by the degree of disorder) will compete with the mode of nonlinear oscillations to influence the interaction between the quasimodes of RL, resulting in non-trivial dynamical transition, i.e., RSB [1, 3]. In 2011, Ref. [6] reported the influences of temperature and amount of disorder on the phase transition in various nonlinear disordered systems through numerical simulation. However, in the field of RL, the influences of temperature and disorder on RSB in RL have not been proved experimentally. In RL, the degree of disorder can be controlled via varying the morphology of the disordered gain system as the previous reports [17–19]. As a result, various fiber structures can be used to tune the degree of disorder in RL based on optical fiber. In 2020 [20], X. Bian et al. show the ring-shaped RL via coating the random gain layer on the surface of fiber, which develops a simple approach to fabricate RL based on optical fiber. Especially for polymer fibers, various fiber structures can be easily tailored to fabricate PFRL due to their tunability and easy manufacture [21–23]. It provides an ideal platform for statistical research in RL.

In this work, temperature and various fiber structures are introduced into the PFRL to analyze their influences on RSB, which indicates that the RSB transition can be tuned by temperature and structure. And the tunable mechanism of temperature and fiber structure on RSB is investigated both experimentally and theoretically, which demonstrates that temperature and disorder are key factors affecting RSB in RL. Meanwhile, we employ the correlation coefficient to analyze the influence of disorder on the interaction between RL modes to support the findings.

## 2 Results and discussion

### 2.1 Polymer fiber random laser

**Figure 1:**
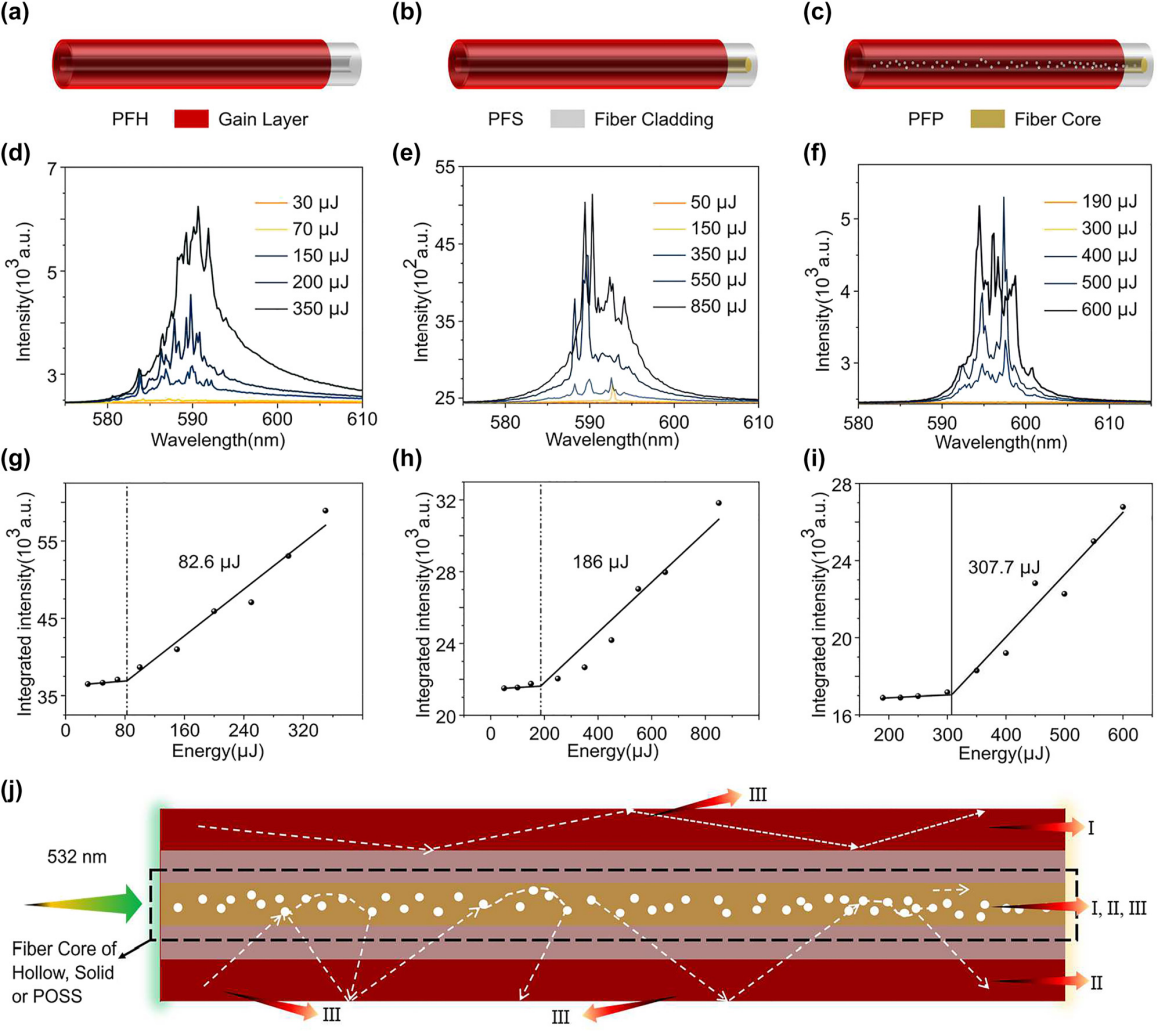
The optical properties of PFRL. (a–c) Schematic of (a) PFH, (b) PFS and (c) PFP, where the white spots in the fiber core of PFP represent the randomly distributed scattering medium. (d–f) The emission spectra of PFH, PFS and PFP, respectively. (g–i) The corresponding integrated emission intensity versus pumping energy. (j) The emission mechanism of PFRL, where I, II and III are the gain paths of the first, second and third parts of seed photons, respectively.

Three types of polymer fibers are introduced to investigate the influence mechanism of temperature and fiber structure on the RSB in RL, including the hollow-core (PFH), solid-core (PFS) and POSS-core (PFP) polymer fibers (see Figure 5). As shown in Figure 1(a)–(c), the Pyrromethene 597 (PM597, Exciton, USA) as the gain medium is coated on the outer surface of polymer fibers with different fiber structures to form gain layer (red coating, thickness ∼1 μm) of PFRL, where the gray represents the fiber cladding (diameter ∼450 μm), yellow represents the fiber core (diameter ∼20 μm) and white spots represent the polyhedral oligomeric silsesquioxanes (POSS) scattering nanoparticles (NPs). As shown in Figure 1(a), PFH has a hollow structure without a fiber core, while PFS contains a solid fiber core but no randomly distributed scattering medium as shown in Figure 1(b). In Figure 1(c), POSS (Hybrid, Hattiesburg, USA) NPs (size 0.5 nm) are doped into the fiber core as a scattering medium, to provide a stronger degree of disorder for PFP than PFS. The emission spectra of the PFH at different pump energy are measured in Figure 1(d). A broad spontaneous emission is observed at a low pump energy and then the multi-mode spike peaks begin to emerge as the energy increased over threshold. Meanwhile, Figure 1(g) shows the dependence of the integrated emission intensities extracted from the corresponding emission spectra on the pump energy, where the threshold behavior of 82.6 μJ is observed in the PFH. It suggests that the PFRL fabricated by coating gain layer on the surface of polymer fiber is observed in PFH. In addition, RL is observed in PFS Figure 1(e) and (h) and PFP Figure 1(f) and (i) with thresholds of 186 μJ and 307.7 μJ, respectively. It illustrates that coating the gain layer on the surface via the pull-up method allows the simple construction of a PFRL.

For the PFRL system involved in this work, the gain layer is pumped to realizes population inversion and randomly radiates seed photons with various directions. The detailed experimental setup can be seen in Figure 6. These seed photons will undergo three processes to participate in the emission of fluorescence and RL in the polymer fiber containing fiber core as shown in Figure 1(j). The first part of the seed photons realize gain due to the feedback provided by the waveguide at the interface of the gain layer and the fiber cladding, which to lase when gain is greater than loss. It is similar to the silicon-based waveguide in polymer film RL [24].The second part of the seed photons are firstly coupled into the core of polymer fiber to undergo multiple scattering from various fiber structures. Finally, it will be sent as feedback to the gain layer to obtain sufficient gain to lase. Where the intensity of multiple scattering is attributed to the degree of disorder provided by the various fiber structures. The rest of the seed photons will be radiated into the air through various paths to achieve fluorescence emission. For PFH without fiber core, the RL is primarily caused by the stimulated emission of the first part of the seed photons, while the RL in PFS and PFP is derived from the coupled RL of the first and second parts. Compared to PFS, the second part of the seed photons in PFP undergoes more disordered multiple scattering due to the presence of POSS NPs in the fiber core. As a result, the degree of disorder in PFS is weaker than in PFP but stronger than in PFH without a core. These polymer fibers with different structures provide a platform to investigate the structurally tunable RSB in PFRL.

### 2.2 Replica symmetry breaking

The RSB transition is characterized by the variation of the statistical distribution of Parisi overlap parameter which is associated with the system replica and characterizes the interaction of disorder and fluctuation in complex system. In the case of RL system, the theoretical replica is related with the amplitudes of the laser modes from the same RL system under the same pump energy in each shot. However, the phase information of the laser modes is not easy to be obtained in the experiments, which hinders the evaluation of the laser modes amplitudes. The only experimentally accessible information of the laser modes is the intensity magnitudes. Thus, the real replica in RL system is defined as the lasing spectrum containing the laser modes intensities $I_{j}\propto {\left|a_{j}\right|}^{2}$ , where *a*_*j*_ is the amplitude of the longitudinal mode *j*. Under each pump energy, we measure *N*_*s*_ system replicas (i.e., laser spectra). The overlap parameter *q*, an analogy to the Parisi parameter, is introduced and calculated as

$$q\alpha \beta =\frac{\overset{N}{\underset{K=1}{\sum }}\Delta \alpha \left(k\right)\Delta \beta \left(k\right)}{\sqrt{\overset{N}{\underset{K=1}{\sum }}\Delta_{\alpha }^{2}\left(k\right)}\sqrt{\overset{N}{\underset{K=1}{\sum }}\Delta_{\beta }^{2}\left(k\right)}}$$

where *α*, *β* = 1, 2 …, *N*_*s*_ are the different replica indexes and $\Delta_{i}\left(k\right)$ is the intensity fluctuation of the *i*th replica at the wavelength index *k*. $\Delta_{i}\left(k\right)$ is given by the equation $\Delta_{i}\left(k\right)=I_{i}\left(k\right)-\bar{I_{i}}\left(k\right)$ . The total number of the overlap parameter *q* is *Ns*(*Ns*−1)/2. Then, the probability density distribution *P*(*q*) of the overlap parameters is analyzed to determine the system regime.

### 2.3 Temperature tunable RSB

**Figure 2:**
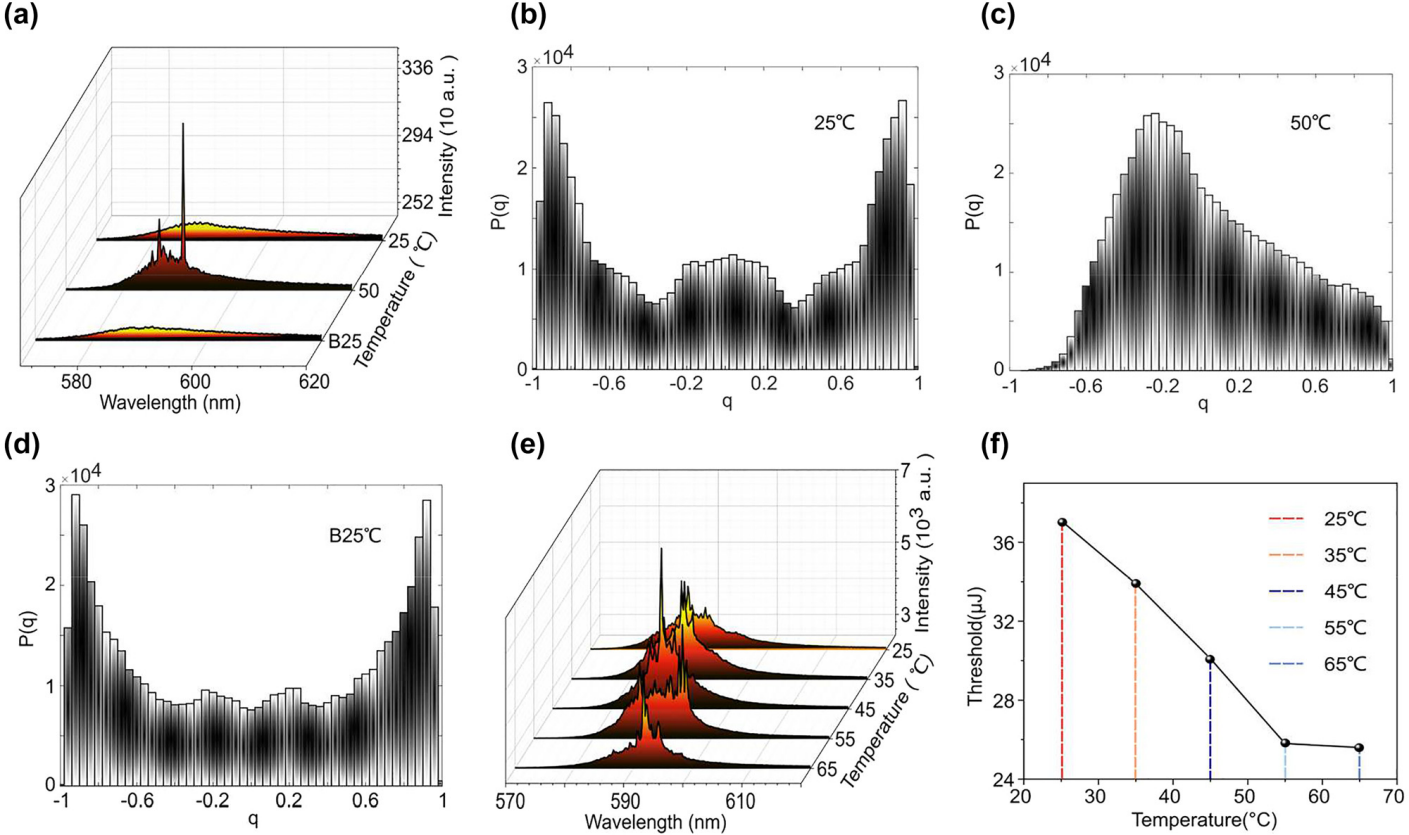
The temperature tunable RSB in PFP. (a) The emission spectra under different temperatures at the pump energy of 35 μJ. (b–d) The probability density distribution of the *q* under 25 °C, 50 °C and B25 °C, respectively. Where the B25 °C demonstrates that cooling from 50 °C to 25 °C. (e) The emission spectra at different temperatures under the pumped energy of ∼60 μJ. (f) The thresholds of RL at different temperatures.

We first investigate the RSB transition in the PFP at the pump energy of 35 μJ. The temperature tunable RSB in PFP is investigated in Figure 2. 1100 emission spectra are collected at each temperature. The wavelength index *k* is in the range between 582.14 nm and 594.45 nm, which contains the spikes of RL in Figure 2(a). Figure 2(b)–(d) shows the distributions *P*(*q*) of the overlap parameters calculated from emission spectra under different temperatures at the pump energy of 35 μJ. In Figure 2(b), the distribution *P*(*q*) shows the boundary of *q* = ±1 with two humps, i.e., spin-glass phase [7]. Where all quasimodes start to oscillate and frustrated by the disorder, which results in the non-trivial overlap distribution. It demonstrates that PFH belongs to the spin-glass system at room temperature at this time. Meanwhile, it was found that the distribution *P*(*q*) are centered around *q* = 0 (spin-glass phase) with the temperature increase to 50 °C, as shown in Figure 2(c). This indicates that all the quasimodes are independent, corresponding to the RL system in an uncorrelated paramagnetic phase. As the temperature cools back to 25 °C, it is interesting that the spin-glass phase transferred back from the paramagnetic phase is observed again in Figure 2(c). In short, the tunable phase transition, i.e., tunable RSB, controlled by temperature is observed in PFRL. According to the properties of spin-glass systems, it exhibits the properties of general magnetic substances at high temperatures, i.e., paramagnetic phase, whereas it exhibits spin-glass phase at low temperatures. This could be responsible for the temperature controlled RSB at the macroscopic level.

To analyze the temperature control mechanism of RSB at micro level, the emission spectra of RL under different temperatures are investigated in Figure 2(e). It is clearly that the wavelength and intensity of the RL spectra change significantly with temperature. In other words, the temperature control mechanism of RSB can be obtained via analyzing the factors influencing the wavelength and intensity. At first, the intensities of the spectra become stronger as the temperature rises from 25 °C to 45 °C. But then that of the spectra no longer increase at 55 °C and 65 °C. The fluctuation in spectral intensity can be attributed to the temperature-dependent change in the refractive index of polymer fibers. In Figure 2(e), at the room temperature, the main peak wavelength is 586.4 nm. With the increase of temperature to 55 °C, the peak wavelength red shifts to 592.74 nm, which red shifts ∼6.4 nm with increasing temperature by 30 °C. In our previous work [25], we demonstrate that increasing temperature will decrease the refractive index of polymer fiber, which further enhance the value of the scattering mean free path. While the long scattering mean free path can lead to a redshift of RL wavelengths. When the temperature increases to 65 °C, the change of wavelength between that under 55 °C and 65 °C can be ignored. It can be attributed to that the influence of temperature on the refractive index of polymer fiber has a transition inflection point around 55 °C [25]. This demonstrates that temperature-induced changes in the refractive index of polymer fibers are the reason of producing the spectrum fluctuation of RL. Changes in temperature, in particular, can influence the degree of disorder in RL systems by modifying the refractive index [26]. As a result, temperature-induced changes in the degree of disorder in polymer fibers can explain temperature tunable RSB in PFRL at the microscopic level.

To further investigate the temperature tunable RSB, the effect of temperature on the threshold of PFP is analyzed in Figure 2(f). When the temperature increases from 25 to 65 °C, the threshold for PFP increase by 11.5 μJ from 37.1 to 25.6 μJ. It demonstrates that the thresholds of PFP are negative linear with the temperatures. Particularly, the threshold no longer decreases when the temperature rises to 65 °C, which is similarly to the results of Figure 2(e). So far, the tunable RSB can be explained based on the influence mechanism of temperature on the threshold of PFP. According to the previous work, the RSB only appears near the threshold and the RL system is in spin-glass phase. In Figure 2(a), at first, the pump energy of 35 μJ is near the PFP threshold at room temperature, but then the pumping energy value becomes higher than the new PFP threshold due to the decrease of the threshold when the temperature rises to 50 °C. The system transitions from the spin-glass phase to the paramagnetic phase when the pump energy exceeds the threshold. It demonstrates that the effect of temperature on the threshold of RL also allows to explain the tunable RSB in PFRL at the macroscopic level.

As a results, the temperature tunable RSB in PFRL can be explained macroscopically through the effect of temperature on the nature of spin-glass system and the RL threshold. And the influence of temperature on the degree of disorder via tuning the refractive index can explain the temperature tunable RSB microscopically.

### 2.4 Structurally tunable RSB

**Figure 3:**
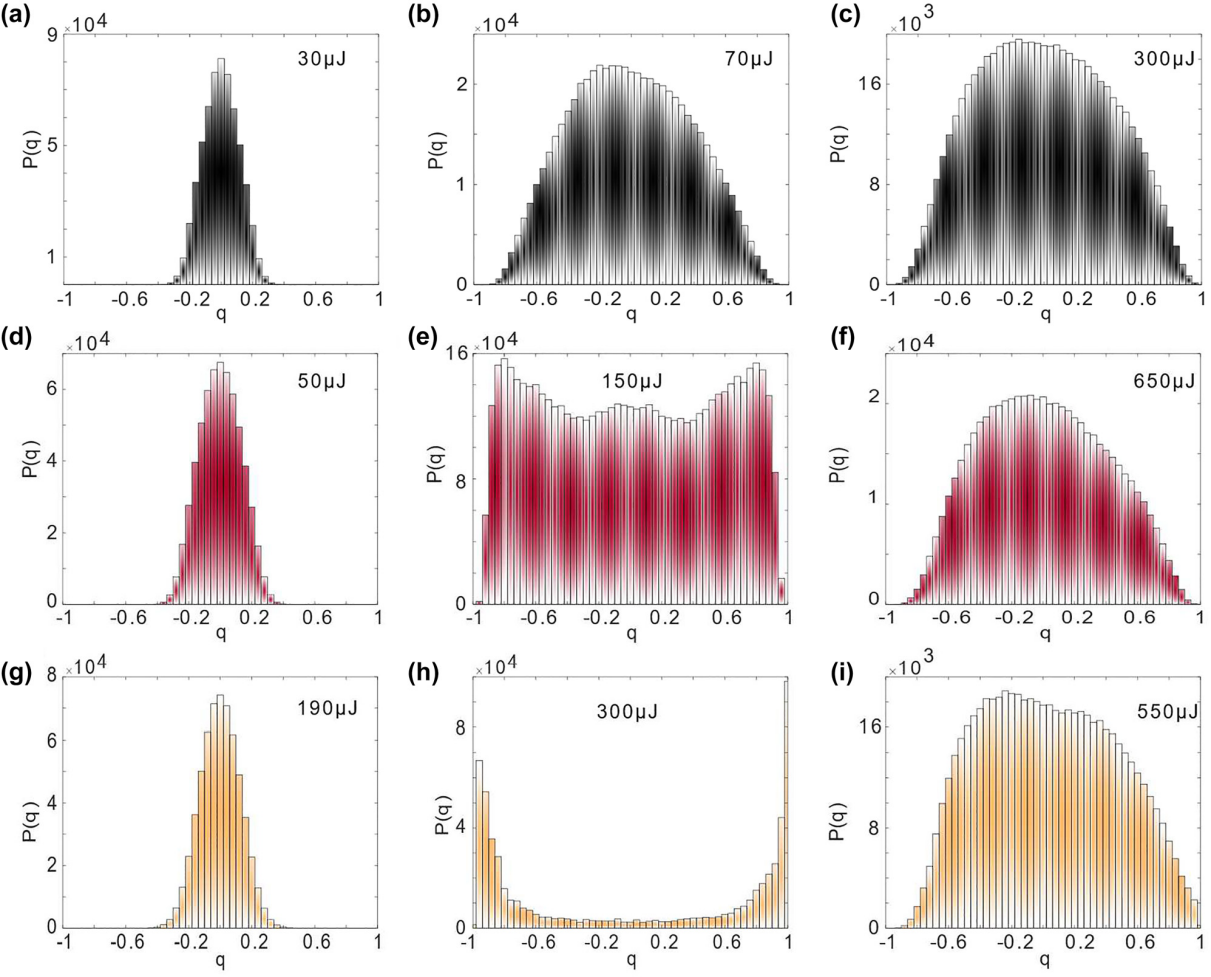
Overlap distributions signalizing the RSB transition. (a–i) Plot of *P*(*q*) versus *q* for different pumping energy from below (a, d and g) to above (c, f and i) and around the threshold (b, e and h) for PFH (the first row), PFS (the second row) and PFP (the third row).

The RSB in PFH, PFS and PFP is investigated in Figure3 to further analyze the influence mechanism of disorder on RSB in PFRL. 1100 emission spectra are collected at each pump energy to calculate the probability distribution *P*(*q*) of the Parisi overlap parameter *q*. Figure 3 shows the *P*(*q*) distributions of the overlap parameters calculated from spectral intensities at different pump energy. For PFH, it is found that the *P*(*q*) distribution remains centered around *q* = 0 from below to above the laser threshold of 82.6 μJ, as shown in Figure 3(a)–(c). It indicates that the PFH is in paramagnetic phase [7]. But in Figure 3(e), around the threshold of 186 μJ, the *P*(*q*) distribution broadens and finally reaches the boundary of *q* = ±1 with two humps, which suggests that the PFS is in spin-glass phase. And the PFS is all in paramagnetic phase below and above the threshold [11] as shown in Figure 3(d) and (f). The transition from paramagnetic phase to spin-glass phase is a typical feature of RSB, although the RSB phenomenon in PFS is weakly. Furthermore, the variation between paramagnetic phase and spin-glass phase is clearly observed in PFP as shown in Figure 3(g)–(i). It is interesting that the humps distribution in PFP is more obvious than that in PFS around the threshold.

The different statistical analysis phenomena are observed in PFH, PFS and PFP, which indicates that the structure of polymer fiber can influence the RSB in PFRL. Figure 1 demonstrates that the degree of disorder in PFS is stronger than in PFH but weaker than in PFS. According to the spin-glass theory in RL, RSB occurs when the disorder in RL is strong enough to compete with the nonlinearity [14]. For PFH, RL mainly originates from the stimulated oscillation of the first part of photons. The modes is independent and do not interact below the threshold [27]. With increasing energy, RL modes start to oscillate coherently under nonlinear action due to the absence of disorder, which exhibits the properties of general magnetic systems (non-spin-glass) [6]. In PFP, localized modes formed by strong disorder will compete with the coherently oscillating modes, which makes the interaction between RL modes to become disordered. In other words, the coherently oscillating modes are frustrated by disorder [7], resulting in spin-glass phase, as shown in Figure 3(h). However, the coherently oscillating modes in PFS cannot be effectively frustrated as the fiber core cannot provide enough disorder to compete with nonlinearity. As a result, the incomplete transition of RSB is observed in PFS as shown in Figure 3(e). The structurally tunable RSB is observed in PFRL as a result of the different degrees of disorder induced by various fiber structures.

**Figure 4:**
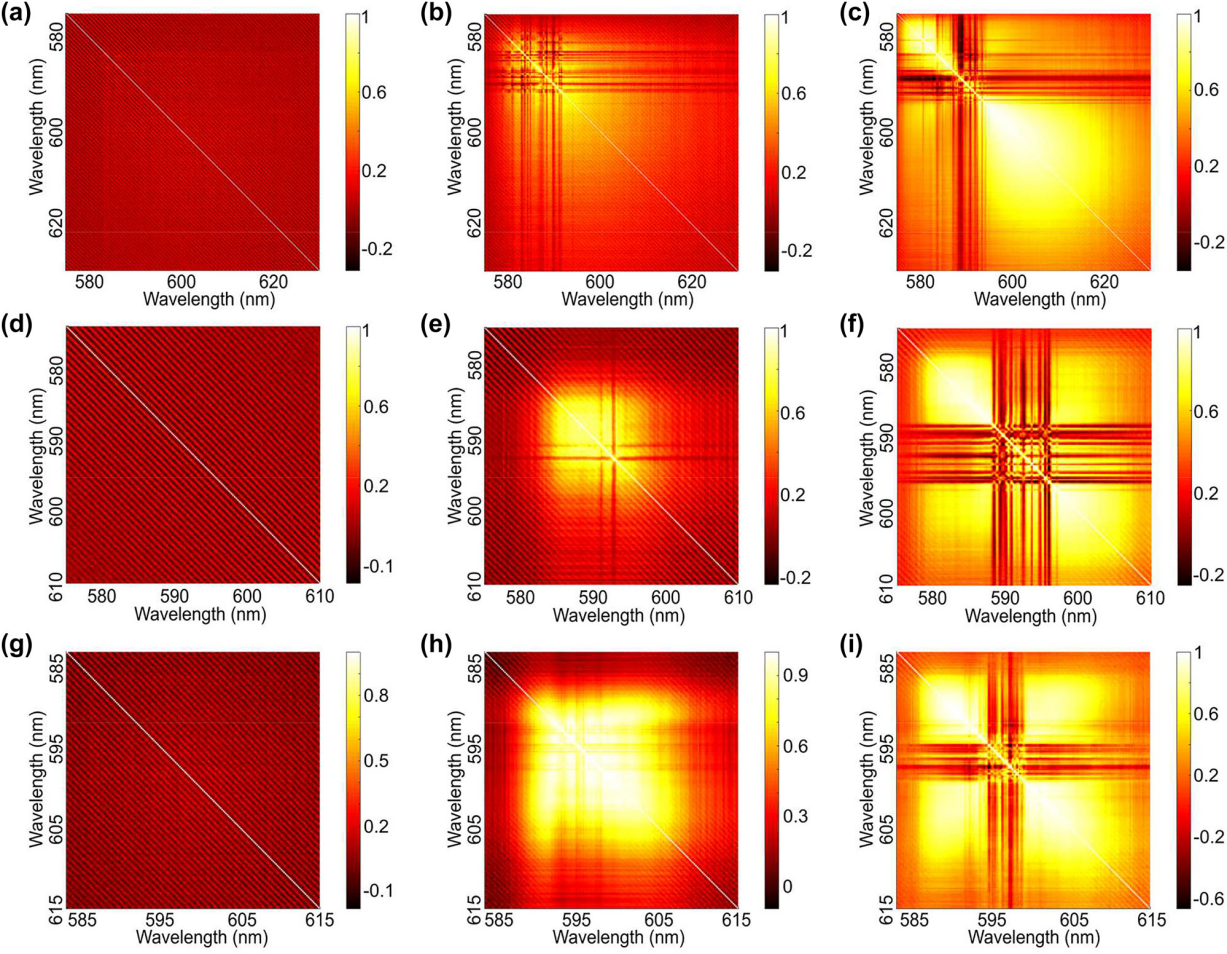
Correlation coefficient heatmaps of the spectral intensities across the RL quasimodes. (a–i) The correlation coefficient heatmaps are evaluated from the spectral intensities for PFH (the first row), PFS (the second row) and PFP (the third row) below (a, d and g), above (c, f and i) and around the threshold (b, e and h), which corresponding the pump energy in Figure 3.

To prove the accuracy of the RSB phenomenon in PFRL in Figure 3, the explicit correlations between lasing mode intensities for all samples are quantified using the correlation coefficient [28, 29] in Figure 4. The correlation coefficient can be expressed at two different wavelengths *λ*_1_ and *λ*_2_ *.* Meanwhile, the correlation coefficient $r\left(\lambda_{1},\lambda_{2}\right)$ can be calculated as

$$r\left(\lambda_{1},\lambda_{2}\right)=\frac{c\left(\lambda_{1},\lambda_{2}\right)}{\sqrt{v\left[\lambda_{1}\right]v\left[\lambda_{2}\right]}}$$

where $v\left[\lambda_{1}\right]$ and $v\left[\lambda_{2}\right]$ are the variance of *λ*_1_ and *λ*_2_, the covariance between *λ*_1_ and *λ*_2_ can be expressed as $c\left(\lambda_{1},\lambda_{2}\right)$ . In the plot of correlation coefficient, the diagonal elements represent the autocorrelations of individual modes, while the off-diagonal elements are the cross-correlation across the modes.

As shown in Figure 4(a), (d) and (g), the auto-correlations are strong and there are no cross-correlations between quasimodes, which indicates that the quasimodes are independent and do not interact [19]. It is consistent with the paramagnetic phase in Figure 3(a), (d) and (g). For PFH, the cross-correlations between the RL modes are observed in Figure 4(b) around the RL threshold. But the positive correlations are so weakly that the mode coupling appears weak [30]. Meanwhile, although both the positive correlations and negative correlations are enhanced with the energy increasing to 300 μJ, the distribution of cross-correlations is still discrete with no significant transitions between Figure 4(b) and (c). Around the threshold for PFS, Figure 4(e) shows that the strong positive correlation and weak negative correlation between quasimodes are uniformly distributed, indicating that non-trivial resonance coupling (spin-glass phase) between most quasimodes appears as the pump energy increased. It differs significantly from the discrete distribution (paramagnetic phase) of the cross-correlation in Figure 4(b). As the pump energy increases above the threshold, the cross-correlations are broken as shown in Figure 4(f), which becomes a discrete distribution similar to that in Figure 4(c). For PFS, the significant transition is observed in the correlation coefficient heatmap from below to above the threshold, which is consistent with the RSB phenomenon in P FS. Similarly, the transition from uniform to discrete distribution in the correlation coefficient heatmap is also observed in PFP as shown in Figure 4(h) and (i). It is consistent with the transition between the paramagnetic and spin-glass phases observed in PFP. Comparing to PFS, however, the homogeneously positive correlations without negative correlations around the threshold is observed in Figures 4(h), which demonstrates that all quasimodes in PFP exhibit non-trivial correlations. It is responsible for the more complete RSB transition observed in PFP than in PFS. As a results, we prove the accuracy of the RSB phenomenon in Figure 3 by analyzing the correlation coefficient between modes of PFRL.

The statistical analysis results observed in PFRL of different structures are shown in Table 1, which are introduced to analyze the influence of the fiber structure on the RSB. In Table 1, it is concluded that RSB cannot be observed in the PFRL system without a core by comparing PFH with PFS and PFP, which indicates that the core is the key factor that affects the RSB phenomenon in PFRL. Meanwhile, the strong RSB phenomenon can be observed in PFP with a strongly disordered core, which can be concluded by comparing PFS observed weak RSB phenomenon. Where the POSS NPs allows to provide strong disorder to enhance the multiple scattering in RL system. As a result, Table 1 indicates the RSB in PFRL can be controlled by fiber structure.

**Table 1:** The statistical analysis results for PFH, PFS and PFP.

Name	Types	Fiber core	RSB
PFH	Hollow-core	N	N
PFS	Soild-core	W	W
PFP	Poss-core	Y	Y

Types: the core structure of polymer fibers; N in fiber core: no core; W in fiber core: weakly disordered core; Y in fiber core: strongly disordered core; N in RSB: no RSB phenomenon; W in RSB: weak RSB phenomenon; Y in RSB: strong RSB phenomenon.

## 3 Conclusions

In summary, the temperature and structure tunable RSBs in PFRL are realized in this work, which provides the experimental evidence for the tunable RSB in RL. For temperature tunable RSB, the influence of temperature on the RL spectra and threshold are analyzed to explore the influence mechanism of temperature on RSB in PFRL. It reveals that the influence of temperature on the intrinsic properties of spin-glass systems and RL systems can be macroscopically responsible for the temperature tunable RSB in PFRL, while the temperature-induced changes in the degree of disorder in polymer fibers can microscopically explain the temperature tunable RSB in PFRL. Meanwhile, PFRL systems with different fiber structures are compared to analyze the structure tunable RSB. It indicates that structure-induced changes in the degree of disorder in polymer fibers can be responsible for the structure tunable RSB in PFRL. However, the influence mechanism of the disorder on the RSB in RL needs further work to explore. The tunable RSB in PFRL not only provides a more accurate theoretical basis to support RSB based statistical analysis in RL, but also broadens the direction to investigate the rich physical mechanisms involved in RL via spin-glass theory.

## 4 Materials

**Figure 5:**
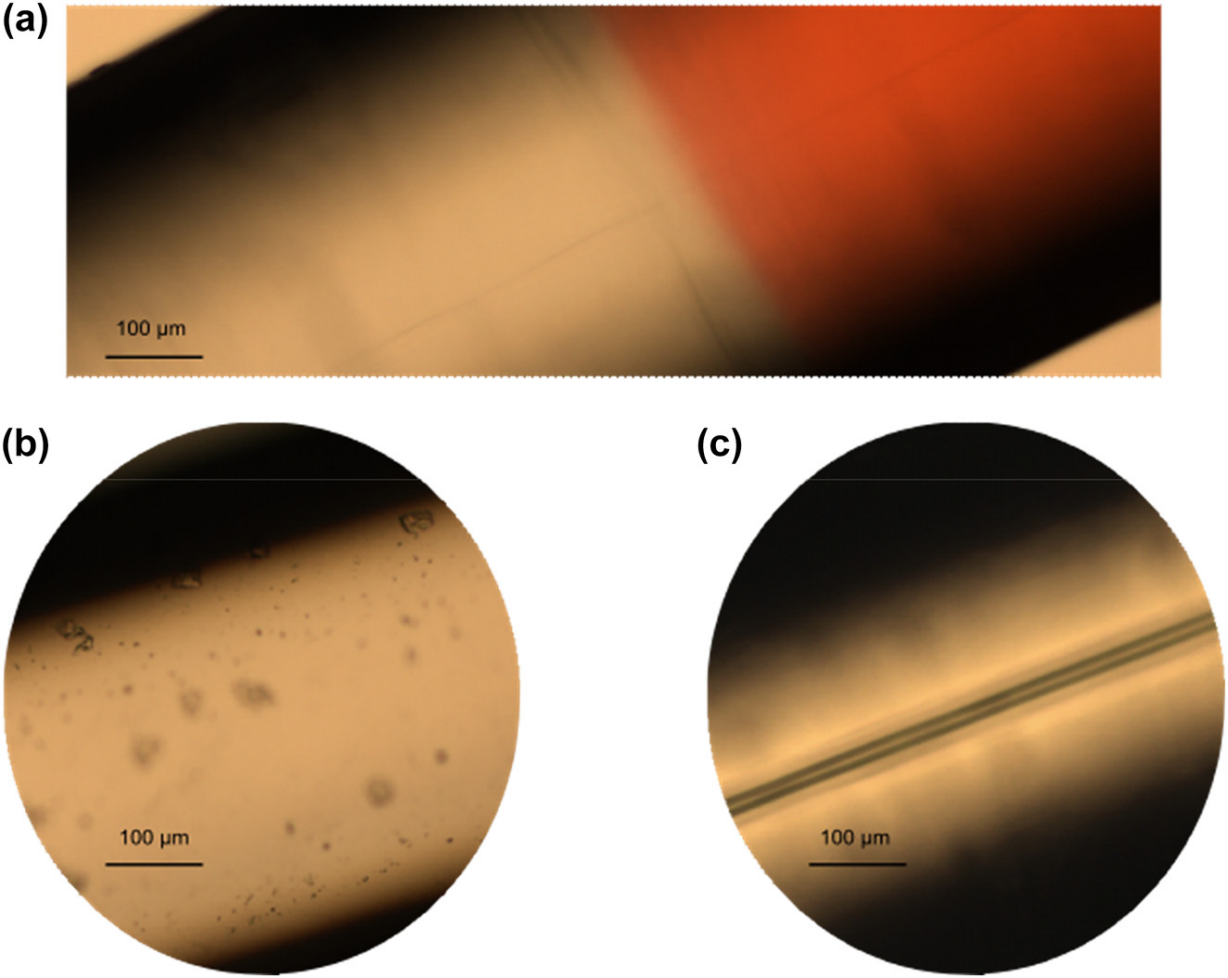
The optical microscope images of PFP, PFS and PFH. It corresponds to (a) POSS-core, (b) solid-core and (c) hollow-core polymer fibers, respectively. Where the red part of PFP is the gain layer coated on the surface of polymer fiber, with a thickness of about 1 μm.

Three types of dye-coated polymer fibers are introduced to investigate the tunable RSB in RL. First, the coating solution is fabricated via dissolving 33.4 wt% poly(methyl methacrylate) (PMMA, Aladdin, Shanghai, China) in 0.3 wt% PM597-doped dichloromethane (CH_2_Cl_2_, SCR, Shanghai, China). Second, all the polymer fibers are fabricated according to the ‘Teflon Technique’ [31, 32], with the diameter ∼450 μm as shown in Figure 5. Where the cladding materials of all the polymer fibers are poly(methylmethacrylate-*co*-butyl acrylate) [poly(MMA-*co*-BA)]. The hollow-core polymer fiber (PFH) is only fabricated by the cladding material as shown in Figure 1(a). The host core materials of the solid-core (PFS) polymer fiber is poly(methylmethacrylate-*co*-benzylmethacrylate) [poly(MMA-*co*-BzMA], and that of the POSS-core (PFP) is poly(methylmethacrylate-*co*-benzylmethacrylate-*co*-methacrylisobutyl polyhedral oligomeric silsesquioxanes) [poly(MMA-*co*-BzMA-*co*-MMAPOSS, where the concentration of POSS NPs is 29 wt%. Finally, the coating solution is uniformly coated on the surface of POSS-, solid- and hollow-core polymer fibers via Pull-Up method, and then volatilized at room temperature to solidify into PFH, PFP and PFS, respectively. In Figure 5(a), the red part is the POSS-core polymer fiber coated with coating solution, and a thin POSS core of PFP can be clearly observed on its axis. To clearly show the core structure of the polymer fiber, the solid-core and hollow-core polymer fibers are not coated by coating solution in Figure 5(b) and (c). Where the core of the solid polymer fiber is not observed in the microscope due to the similar chemical properties of the core and cladding materials. Meanwhile, the hollow core can be observed in Figure 5(c), which is the most obvious difference from PFP.

**Figure 6:**
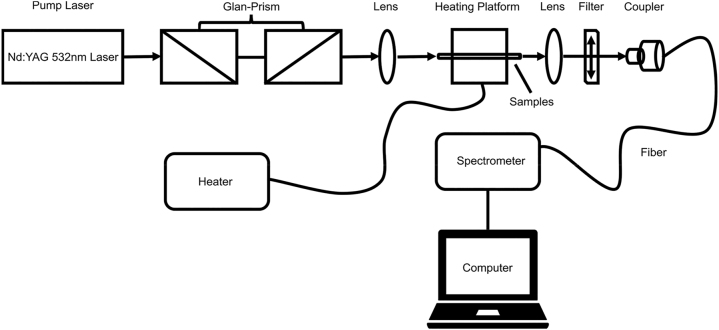
Measurement setup for all the samples involved in the experiment. Where the sample is pumped parallel to the fiber axis by the pump laser and the RL is collected by the spectrometer at the other end. The length of the polymer fiber sample is about 30 mm.

Figure 6 shows the measurement and control setup of the PFRL. A Q-switched Nd:YAG laser (Q-smart 850, Quantel, France), which has an output wavelength of 532 nm with a round spots (pulse duration of 10 ns, repetition rate of 10 Hz, spot diameter of 100 μm), is used to end pump the different types of samples with convex lens (*D* = 25 mm, *f* = 10 cm). Pump pulse energy and polarization are controlled by a Glan Prism group. The emitted light is collected through lens and filter by a fiber spectrometer (QE65PRO, Ocean Optics, resolution of ∼0.4 nm, integration time of 100 ms), where the filter is used to remove excess 532 nm laser. For the process of the temperature control, the sample is placed on the heating platform to achieve real-time temperature control for PFP and the real-time temperature can be displayed directly on the heater screen.
